# A graph-theory method for pattern identification in geographical epidemiology – a preliminary application to deprivation and mortality

**DOI:** 10.1186/1476-072X-8-28

**Published:** 2009-05-13

**Authors:** Ravi Maheswaran, Cheryl Craigs, Simon Read, Peter A Bath, Peter Willett

**Affiliations:** 1 Public Health GIS Unit, School of Health and Related Research, University of Sheffield, Regent Court, 30 Regent Street, Sheffield, S1 4DA, UK; 2 Department of Information Studies, University of Sheffield, Western Bank, Sheffield, S10 2TN, UK

## Abstract

**Background:**

Graph theoretical methods are extensively used in the field of computational chemistry to search datasets of compounds to see if they contain particular molecular sub-structures or patterns. We describe a preliminary application of a graph theoretical method, developed in computational chemistry, to geographical epidemiology in relation to testing a prior hypothesis. We tested the methodology on the hypothesis that if a socioeconomically deprived neighbourhood is situated in a wider deprived area, then that neighbourhood would experience greater adverse effects on mortality compared with a similarly deprived neighbourhood which is situated in a wider area with generally less deprivation.

**Methods:**

We used the Trent Region Health Authority area for this study, which contained 10,665 census enumeration districts (CED). Graphs are mathematical representations of objects and their relationships and within the context of this study, nodes represented CEDs and edges were determined by whether or not CEDs were neighbours (shared a common boundary). The overall area in this study was represented by one large graph comprising all CEDs in the region, along with their adjacency information. We used mortality data from 1988–1998, CED level population estimates and the Townsend Material Deprivation Index as an indicator of neighbourhood level deprivation. We defined deprived CEDs as those in the top 20% most deprived in the Region. We then set out to classify these deprived CEDs into seven groups defined by increasing deprivation levels in the neighbouring CEDs. 506 (24.2%) of the deprived CEDs had five adjacent CEDs and we limited pattern development and searching to these CEDs. We developed seven query patterns and used the RASCAL (Rapid Similarity Calculator) program to carry out the search for each of the query patterns. This program used a maximum common subgraph isomorphism method which was modified to handle geographical data.

**Results:**

Of the 506 deprived CEDs, 10 were not identified as belonging to any of the seven groups because they were adjacent to a CED with a missing deprivation category quintile, and none fell within query Group 1 (a deprived CED for which all five adjacent CEDs were affluent). Only four CEDs fell within Group 2, which was defined as having four affluent adjacent CEDs and one non-affluent adjacent CED. The numbers of CEDs in Groups 3–7 were 17, 214, 95, 81 and 85 respectively. Age and sex adjusted mortality rate ratios showed a non-significant trend towards increasing mortality risk across Groups (Chi-square = 3.26, df = 1, p = 0.07).

**Conclusion:**

Graph theoretical methods developed in computational chemistry may be a useful addition to the current GIS based methods available for geographical epidemiology but further developmental work is required. An important requirement will be the development of methods for specifying multiple complex search patterns. Further work is also required to examine the utility of using distance, as opposed to adjacency, to describe edges in graphs, and to examine methods for pattern specification when the nodes have multiple attributes attached to them.

## Background

Geographical epidemiology has a long history [1]. One of the areas of interest in recent years has been the detection of clusters of disease, and more generally of clustering of disease, and numerous methods have been developed to examine these aspects [2]. These approaches however do not start with a hypothesis but examine disease patterns and lead to hypothesis generation.

An alternative approach is to decide a priori on some geographical pattern of interest based on a hypothesis, and then examine disease rates in areas with and without those patterns to see if they are consistent with the prior hypothesis. Standard geographic information systems (GIS) software may be used to identify such patterns and then to investigate the health experience of populations in these areas of interest. However, if the pattern is complex, then specifying the pattern of interest could also become quite complex within standard GIS packages.

An alternative approach which may offer some potential is the use of methods based on graph theory. Graph theoretical methods are extensively used in the field of computational chemistry to search datasets of compounds to see if they contain particular molecular sub-structures or patterns. We have previously described an adaptation of these methods for use with geographical datasets to identify pre-specified geographical patterns [3-5].

In this paper, we describe a preliminary application of this methodology to geographical epidemiology in relation to testing a prior hypothesis. We tested the methodology on the hypothesis that if a socioeconomically deprived neighbourhood is situated in a wider deprived area, then that neighbourhood would experience greater adverse effects on mortality compared with a similarly deprived neighbourhood which is situated in a wider area with generally less deprivation.

## Methodology

### Geographical area and data

We used the Trent Region Health Authority area for this study. It had a population of approximately 5 million people. We used census enumeration districts (CED) as a proxy for neighbourhood areas, of which there were 10,665 in the Trent Region. CEDs were the lowest level of 1991 census geography at which detailed population information was available in England and Wales.

Mortality data were provided by the Office for National Statistics and included all deaths from 1988 to 1998. The population denominator data were based on the 1991 CED level mid-year population estimates by five-year age band and sex, corrected for under-enumeration [6]. For years preceding and subsequent to 1991, these counts were scaled using district health authority age and sex specific mid-year estimates also obtained from the Office for National Statistics.

The Townsend Material Deprivation Index was used as an indicator of neighbourhood level deprivation [7]. It is a standardized score using four census variables: unemployment, overcrowding, lack of owner occupied accommodation and lack of car ownership. It was calculated for each CED within Trent, standardized to Trent region as a whole. 195 CEDs could not be allocated a deprivation score because of missing values in one or more of the census variables, generally because of low counts and suppression thresholds built into the census tables [8]. The remaining 10,470 CEDs were assigned to a deprivation quintile based on their Townsend score. A quintile value of 5 indicated those CEDs within the top 20% most deprived areas, and a quintile value of 1 indicated those CEDs within the top 20% most affluent, relative to Trent.

### Graph theory method

Graph theory is a branch of mathematics which is concerned with the study of 'graphs', which are mathematical representations of objects and their relationships. The graph is a collection of points (referred to as nodes or vertices) connected by lines (referred to as edges). The nodes may have various attributes attached to them and the edges may represent adjacency, distance or some other connection between pairs of nodes. Within the context of this study, the nodes represented CEDs and the edges were determined by whether or not CEDs were neighbours (i.e. they shared a common boundary).

The overall area in this study was represented by one large graph comprising all the CEDs in the region, along with their adjacency information. The information relating to the graph was held in a space-separated text file. The file contained three parts. The first part held, on one line, the total number of CEDs, the maximum number of neighbouring CEDs and the number of variables describing attributes attached to the CEDs. The second part held, for each CED, one line containing the CED number, CED code, the deprivation quintile and the number of adjacent CEDs. The third part held, for each CED, one line containing the CED number and the CED number for each adjacent CED.

An extract from the data file is shown below, displaying the format of the text file:

10665   22   2 (Part 1)

.

10000   38PMFF03   4   6 (Part 2)

.

10000 9998 9999 10001 10002 10003 10004 0 0 0 0 0 0 0 0 0 0 0 0 0 0 0 0 (Part 3)

Part 1 shows there were 10665 CEDs within the data file, a possible maximum of 22 adjacent CEDs, and that there were two variables describing attributes (deprivation category and number of adjacent CEDs) for each CED. Part 2 shows that the CED 38PMFF03 was numbered 10000, had a deprivation quintile of 4 and had 6 neighbouring CEDs. Part 3 shows the CED number and the numbers of the six neighbouring CEDs. Because the maximum number of neighbouring CEDs in this dataset was 22, the graph theory based program expected 22 numbers to follow each CED number in part 3. The CED 38PMFF03 had only 6 neighbouring CEDs so 16 zeroes were included to ensure that the CED had the 22 expected values.

For this demonstration study, we defined deprived CEDs as those in the top 20% most deprived in the region, i.e. quintile category 5. We defined CEDs in quintile categories 1 and 2 as affluent CEDs and CEDs in quintile categories 3 and 4 as non-affluent and non-deprived CEDs. We then set out to classify the deprived CEDs into seven groups determined by the deprivation levels in the surrounding areas, using the classification below:

Group 1   All affluent surrounding areas

Group 2   75% – <100% affluent surrounding areas

Group 3   50% – <75% affluent surrounding areas

Group 4   < 50% affluent, <50% deprived

Group 5   50% – <75% deprived surrounding areas

Group 6   75% – <100% deprived surrounding areas

Group 7   All deprived surrounding areas

We then examined the distribution of the 2094 CEDs in quintile category 5 in terms of the number of adjacent CEDs. Table 1 shows the distribution of the number of neighbouring CEDs for these deprived CEDs. The number of neighbours ranged from 2 to 19. For the purpose of this demonstration study, we used the category which contained the highest number of deprived CEDs i.e. deprived CEDs with five adjacent CEDs, of which there were 506 (24.2%).

**Table 1:** Distribution of the number of neighbouring CEDs for 2094 deprived CEDs.

**Number of adjacent CEDS**	**Frequency**	**Percent**
2	27	1.3
3	160	7.6
4	347	16.6
5	506	24.2
6	433	20.7
7	303	14.5
8	125	6.0
9	78	3.7
10	49	2.3
11	31	1.5
12	12	0.6
13	9	0.4
14	6	0.3
15	5	0.2
16	1	0.05
17	1	0.05
19	1	0.05

Total	2094	100

**Figure 1:**
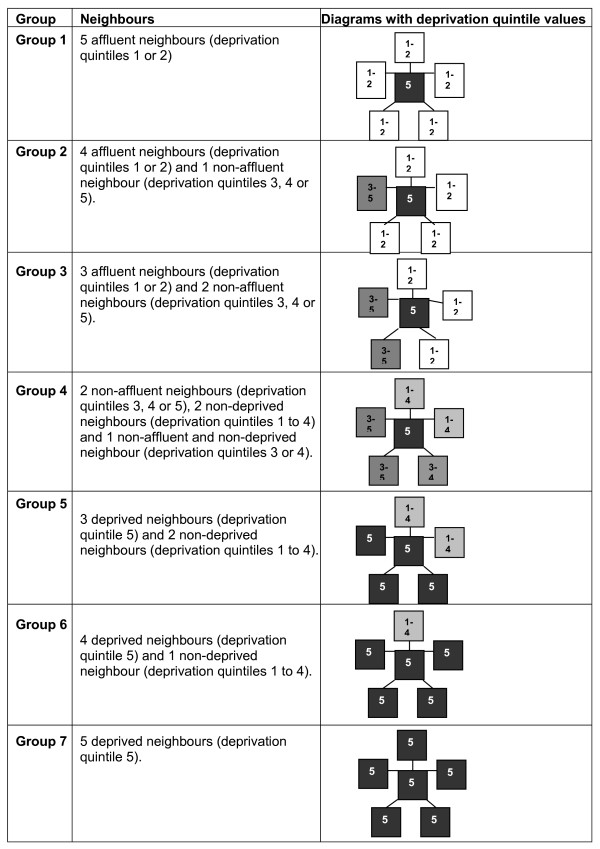
**Query patterns used to identify and assign deprived CEDs with five neighbouring CEDs into seven groups based on deprivation levels in the neighbouring CEDs (see text for definitions of the groups)**.

To operationalise the seven-group classification in relation to five adjacent CEDs, we produced seven query patterns. Figure 1 shows the query patterns used to identify and assign the deprived CEDs with five neighbours into the seven groups based on deprivation levels in the neighbouring CEDs.

We used the RASCAL (Rapid Similarity Calculator) program to carry out the search for each of the query patterns [9]. This program uses a maximum common subgraph isomorphism method which was modified to handle geographical data and used to detect subgraph isomorphism in this demonstration study. This is where the search was set to detect matches where 100% of the edges in the query graph were contained within the data graph.

The version of RASCAL we used contains an algorithm that makes use of a mathematical structure known as a modular product graph to reduce the computational expense (i.e. time) of maximum common subgraph isomorphism detection [10]. Within the modular product graph, each node symbolises a pairing of one node from the CED graph and one node from the query graph. A 'clique' is any set of nodes within a graph that is connected to every other node in the set. A clique in the modular product graph happens to correspond to an edge subgraph common to both the CED and query graph. By finding the maximum clique in the modular product graph, we indirectly find the largest area of common edge overlap between the CED and the query graph. This modular product graph approach is more effective than directly searching for the maximum common subgraph, as more efficient algorithms exist for clique detection.

### Statistical analysis

We grouped the population and death counts by age, sex and seven-category deprivation group. We used Poisson regression analysis in SAS, with correction for overdispersion, to examine the association between deprivation and mortality. We entered age, sex and group (1–7) as categorical variables. Results are presented as rate ratios with 95% confidence intervals.

## Results

The number of deprived CEDs with five adjacent CEDs within each group identified by running the RASCAL program is displayed in table 2. The table shows that of the 506 deprived CEDs, 10 were not identified as belonging to any of the 7 groups. This was because they were adjacent to a CED with a missing deprivation category. These 10 CEDs were not included in any further analysis. The search also showed that none of the CEDs fell within query Group 1, i.e. a deprived CED for which all five adjacent CEDs were affluent. Only four CEDs fell within Group 2, which was defined as having four affluent adjacent CEDs and one non-affluent adjacent CED.

**Table 2:** 506 deprived CEDs with five neighbours categorised by deprivation levels in the neighbouring CEDs.

Group*	**Number of deprived CEDs**
Group 1	0
Group 2	4
Group 3	17
Group 4	214
Group 5	95
Group 6	81
Group 7	85
Not assigned to any group	10

Total	506

* Group 1 – all five neighbouring CEDs were affluent; Group 7 – all five neighbouring CEDs were deprived.

**Figure 2:**
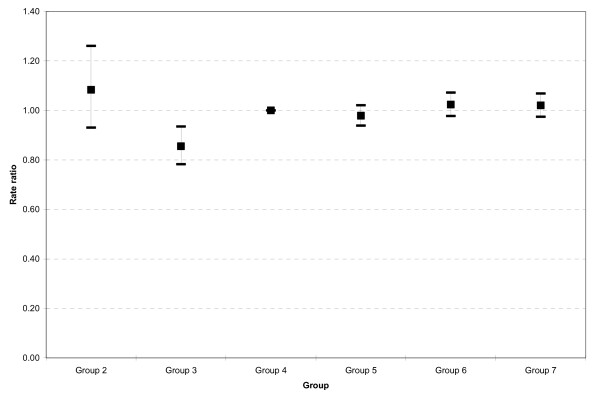
**Age and sex adjusted mortality rate ratios for deprived CEDs with five neighbours categorised by deprivation levels in the neighbouring CEDs**. The groups ranged from Group 1 in which all five neighbouring CEDs were affluent (there were no deprived CEDs in this group) to Group 7 where all five neighbouring CEDs were deprived.

Table 3 shows the deaths, population and age and sex adjusted rate ratios in the CED groups. We used Group 4 as the comparison category as it contained the largest number of CEDs. The adjusted rate ratios are also displayed in figure 2. There was a trend towards increasing mortality risk across groups although this was not significant (Chi-square = 3.26, df = 1, p = 0.07) because Group 2 had a high rate ratio. This group, based on rates from four CEDs, had a single CED with high rates which dominated the rate ratio for the group.

**Table 3:** Deaths, population counts and age and sex adjusted mortality rate ratios for deprived CEDs with five neighbours categorised by deprivation levels in the neighbouring CEDs.

Group*	**Number of deprived CEDs**	**Deaths** **1988–1998**	**Population count**	**Adjusted rate ratio (95% CI)**
1	0	-	-	-
2	4	359	1189	1.08 (0.93 – 1.26)
3	17	1089	8089	0.86 (0.78 – 0.93)
4	214	16315	105424	1.00 (1.00 – 1.00)
5	95	6404	48388	0.98 (0.94 – 1.02)
6	81	4999	39001	1.02 (0.98 – 1.07)
7	85	4947	41519	1.02 (0.97 – 1.07)

* Group 1 – all five neighbouring CEDs were affluent; Group 7 – all five neighbouring CEDs were deprived.

**Figure 3:**
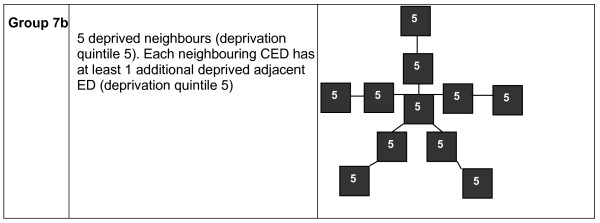
**Additional query used to subdivide Group 7 into two further groups (7a and 7b, where CEDs not identified by the Group 7b query above were assigned to Group 7a)**.

As there were 85 CEDs in Group 7, we then split it into two further groups based on the CEDs surrounding the five adjacent CEDs, i.e. the second order neighbours. We defined Group 7b as the situation where each adjacent CED had at least one deprived adjacent CED. This query pattern is shown in figure 3. We chose this pattern because it was a simple pattern to specify for second order CEDs. CEDs which matched this pattern, of which there were 57, were assigned to Group 7b and the remaining 28 CEDs were assigned to Group 7a. The adjusted rate ratios (relative to Group 4) were 0.92 (0.86 – 0.99) for Group 7a and 1.08 (1.02 – 1.13) for Group 7b.

## Discussion

We found that the basic graph theory method we used to identify neighbourhoods which were surrounded by varying levels of deprivation showed that there was some evidence of a trend towards higher mortality in neighbourhoods surrounded by deprived areas, although this was of borderline significance (p = 0.07). Methodology based on graph theoretical methods available in computational chemistry may be a useful addition to methods available for geographical epidemiology but there are a number of problems which need to be overcome first.

A significant issue is the specification of the search pattern. In our example, we set out to examine mortality rates is deprived CEDs surrounded by CEDs with varying levels of deprivation. For the purpose of this exploratory demonstration project, and to keep the task manageable, we restricted the search patterns and analysis to deprived CEDs with five adjacent CEDs. We chose this category because it was the category with the largest number of deprived CEDs. Even so, the category only comprised 24.2% of deprived CEDs and for a more complete analysis, we would need to specify search patterns for all of the other categories.

Expansion of the search to include deprived CEDs with different numbers of adjacent CEDs raises a further issue. If we have a deprived CED with two adjacent CEDs, both of which are deprived, the effect on the central CED may be less marked than the effect on a CED surrounded by seven deprived CEDs. A further related issue which we have not taken into account is the size of the neighbouring CEDs. The size may be conceptualized in terms of geographical area, the population count or a combination. The graph theory method allows a variety of attributes to be attached to nodes, in this case the CEDs. However, the specification of search patterns which take into account not just the deprivation level but also the population and geographical size would be much more complex than the search patterns we have specified in this demonstration project.

We investigated extending pattern searching to second order neighbours for the deprived CEDs with five deprived neighbours in order to examine the feasibility of specifying second order neighbours. We found that this increased the complexity considerably with a whole range of patterns that could be specified and we settled for a pattern that was simple to specify but which was not very specific. We did not specify the number of second order neighbours the first order CEDs could have in addition to having one second order deprived CED. A complete specification of second order neighbours for our example would have involved specifying a large number of patterns.

We did not find clearer evidence for the hypothesis that a neighbourhood surrounded by deprived neighbourhoods would be more likely to have higher mortality rates than a similarly deprived neighbourhood surrounded by less deprived neighbourhoods. This may be explained partly by the limitations described above, such as not taking the geographical size and population of adjacent CEDs into account when specifying the search patterns. In addition, there are other potential explanations. Our study may not have had sufficient power, particularly as there were no CEDs which fell into Group 1 and only four which fell into Group 2. The latter demonstrated the problem with small numbers where the rate for the group was substantially influenced by the high rate for one of the four CEDs. We used CEDs from the UK 1991 census as proxies for neighbourhoods. Whilst some of these may have been reasonable representations of neighbourhoods, others may well not have been. It could also be argued that the concept of neighbourhoods is a complex issue which is unlikely to be adequately represented by geographical boundaries which are in turn usually defined for other administrative purposes.

The method we have used is an adaptation of search algorithms for 2D graphs, that is, where edges are defined in terms of adjacency. Graph-theoretical methods have been used in computational chemistry for several years and a suite of programs have been developed to address increasingly complex search requirements. These include 3D graphical methods where edges may be defined in terms of distance but we have not as yet explored the utility of 3D graphs and search patterns for geographical epidemiology.

Whilst we have described a preliminary application of graph theory methods within the health geography field, it is important to recognise that these methods have been used in a wide variety of disciplines ranging from physics and mathematics to biology and sociology [11,12]. Conte et al have reviewed the role of graph theory methods in the pattern recognition field, describing taxonomies for the different classes of algorithms and the common types of applications within this field [13]. Within the geographical context, examples of use of graph theory methods include evaluation of landscape connectivity and application to landscape genetics [14,15].

## Conclusion

Graph theoretical methods developed in computational chemistry may be a useful addition to the current GIS based methods available for geographical epidemiology but further developmental work is required. An important requirement will be the development of methods for specifying complex search patterns. Further work is also required to examine the utility of using distance, as opposed to adjacency, to describe edges in graphs, and to examine methods for pattern specification when the nodes (geographical areas) have multiple attributes attached to them.

## Competing interests

The authors declare that they have no competing interests.

## Authors' contributions

RM, PAB and PW jointly conceived the idea for adaptation of the methodology for use in geographical epidemiology. CC carried out the analysis supervised by RM. SR contributed information regarding technical details of the methodology used. RM drafted the paper with contributions from the other authors. All authors read and approved the final manuscript.

## References

[B1] Snow J (1854). On the mode of communication of cholera.

[B2] Maheswaran R, Craglia M, Eds (2004). GIS in Public Health Practice.

[B3] Bath PA, Craigs C, Maheswaran R, Raymond J, Willett P (2002). Validation of graph-theoretical methods for pattern identification in public health datasets. Health Informatics J.

[B4] Bath PA, Craigs C, Maheswaran R, Raymond J, Willett P (2005). Use of graph theory to identify patterns of deprivation, high morbidity and mortality in public health data sets. J Am Med Inform Assoc.

[B5] Bath PA, Craigs C, Maheswaran R, Raymond J, Willett P, Wise S, Craglia M (2008). Pattern identification in public health data sets: the potential offered by graph theory. GIS and Evidence-Based Policy Making.

[B6] Anonymous (1995). Estimating with confidence: mid-1991 population estimates for small areas. Popul Trends.

[B7] Townsend P, Phillimore P, Beattie A (1988). Health and Deprivation: Inequalities and the North.

[B8] Dale R, Marsh C (1993). The 1991 Census user's Guide.

[B9] Raymond JW, Gardiner EJ, Willett P (2002). RASCAL: Calculation of graph similarity using maximum common edge subgraphs. Comput J.

[B10] Raymond JW, Gardiner EJ, Willett P (2002). Heuristics for similarity searching of chemical graphs using a maximum common edge subgraph algorithm. J Chem Inf Comput Sci.

[B11] Newman MEJ (2003). The structure and function of complex networks. Siam Rev.

[B12] May RM (2006). Network structure and the biology of populations. Trends Ecol Evol.

[B13] Conte D, Foggia P, Sasone C, Vento M (2004). Thirty years of graph matching in pattern recognition. Int J Pattern Recogn Artif Intell.

[B14] Minor ES, Urban DL (2008). A graph-theory framework for evaluating landscape connectivity and conservation planning. Conserv Biol.

[B15] Garroway CJ, Bowman J, Carr D, Wilson PJ (2008). Applications of graph theory to landscape genetics. Evol Appl.

